# Characterization of Effector and Memory T Cell Subsets in the Immune Response to Bovine Tuberculosis in Cattle

**DOI:** 10.1371/journal.pone.0122571

**Published:** 2015-04-16

**Authors:** Mayara F. Maggioli, Mitchell V. Palmer, Tyler C. Thacker, H. Martin Vordermeier, W. Ray Waters

**Affiliations:** 1 Infectious Bacterial Diseases of Livestock Research Unit, National Animal Disease Center, Ames, IA, United States of America; 2 Department of Veterinary Pathology, College of Veterinary Medicine, Iowa State University, Ames, IA, United States of America; 3 Tuberculosis Research Group, Animal Health and Veterinary Laboratories Agency-Weybridge, New Haw, Addlestone, United Kingdom; University of Cape Town, SOUTH AFRICA

## Abstract

Cultured IFN-γ ELISPOT assays are primarily a measure of central memory T cell (Tcm) responses with humans; however, this important subset of lymphocytes is poorly characterized in cattle. Vaccine-elicited cultured IFN-γ ELISPOT responses correlate with protection against bovine tuberculosis in cattle. However, whether this assay measures cattle Tcm responses or not is uncertain. The objective of the present study was to characterize the relative contribution of Tcm (CCR7^+^, CD62L^hi^, CD45RO^+^), T effector memory (Tem, defined as: CCR7^-^, CD62L^low/int^, CD45RO^+^), and T effector cells (CCR7^-^, CD62L^-/low^, CD45RO^-^), in the immune response to *Mycobacterium bovis*. Peripheral blood mononuclear cells (PBMC) from infected cattle were stimulated with a cocktail of *M*. *bovis* purified protein derivative, rTb10.4 and rAg85A for 13 days with periodic addition of fresh media and rIL-2. On day 13, cultured PBMC were re-stimulated with medium alone, rESAT-6:CFP10 or PPDb with fresh autologous adherent cells for antigen presentation. Cultured cells (13 days) or fresh PBMCs (*ex vivo* response) from the same calves were analyzed for IFN-γ production, proliferation, and CD4, CD45RO, CD62L, CD44, and CCR7 expression via flow cytometry after overnight stimulation. In response to mycobacterial antigens, ~75% of CD4^+^ IFN-γ^+^ cells in long-term cultures expressed a Tcm phenotype while less than 10% of the *ex vivo* response consisted of Tcm cells. Upon re-exposure to antigen, long-term cultured cells were highly proliferative, a distinctive characteristic of Tcm, and the predominant phenotype within the long-term cultures switched from Tcm to Tem. These findings suggest that proliferative responses of Tcm cells to some extent occurs simultaneously with reversion to effector phenotypes (mostly Tem). The present study characterizes Tcm cells of cattle and their participation in the response to *M*. *bovis* infection.

## Introduction

Bovine tuberculosis (bTB) is a chronic bacterial disease of animals that may also infect humans. *Mycobacterium bovis*, the main agent causing bTB, is a member of the *Mycobacterium tuberculosis* complex, which also comprises: *M*. *tuberculosis* (*M*. *tb*), *M*. *canettii*, *M*. *africanum*, *M*. *pinnipedii*, *M*. *microti*, *M*. *caprae* and *M*. *mungi* [1, 2]. This genetically related group of bacteria causes TB with comparable pathology in a wide variety of hosts [3, 4]. Great strides have been made over the past century in the control of bTB in cattle and to limit the risk to humans (e.g., pasteurization of milk for dairy products); however, the disease persists as a significant socioeconomic hardship for livestock farmers with estimates of >50 million cattle infected worldwide, costing $3 billion annually. The WHO (World Health Organization), in conjunction with FAO (Food and Agriculture Organization of the United Nations) and OIE (Office International des Épizooties), recently classified bTB as a neglected zoonosis.

An essential component of the immune response to TB in humans, cattle and mice is the production of IFN-γ by T helper 1 (Th1) CD4 T cells [5–10]. Immune deficiencies affecting CD4 T cells (e.g., HIV infection) and IL-12/IFN-γ /STAT1 signaling pathways result in more severe disease upon TB infection in humans [11,12]. Given the importance of Th1 cells in the immune response to TB, it is not surprising that IFN-γ release assays (IGRA) and delayed type hypersensitivity (i.e., skin test) responses are useful correlates of infection (reviewed by Schiller *et al*. [13] for cattle and Walzl *et al*. [14] for humans). Widely utilized in IGRAs, early secretory antigenic target-6 (ESAT-6) and culture filtrate protein-10 (CFP-10) are potent inducers of Th-1 cytokines [15]. ESAT-6 and CFP-10 are co-secreted proteins encoded by the RD-1 region of the genome of *M*. *tb* complex mycobacteria. Such genes are absent in all *M*. *bovis* bacillus Calmette Guerin (BCG) strains and most other non-tuberculous mycobacteria species [16–19]. Diagnostic IGRA’s are measures of ‘*ex vivo*’ immune responses relying on rapid production of IFN-γ in response to mycobacterial antigen stimulation in short-term (16–24 h) whole blood or peripheral blood mononuclear cell (PBMC) cultures. *Ex vivo* assays for use in bTB diagnosis are generally considered a measure of T cell effector responses and are frequently used to measure immune responses to bTB vaccines prior to and after challenge with virulent *M*. *bovis* [9,20]. While most protective bTB vaccines elicit *ex vivo* IFN-γ responses, not all vaccines that induce this response provide protection [21]. Additionally, levels of IFN-γ elicited by vaccination do not necessarily correlate with the level of protection afforded by the vaccine [22]. Thus, the identification of correlates of protection is needed to prioritize vaccine candidates for evaluation in costly BL-3 vaccination/challenge efficacy trials.

Recent vaccine efficacy studies in cattle have demonstrated that long-term cultured IFN-γ ELISPOT (so called, cultured IFN-γ ELISPOT) responses are positive predictors of vaccine efficacy [23–25] and duration of immunity [26]. Protection provided by vaccination is partial and protected animals have reduced mycobacterial burden and associated pathology following experimental infection. In this assay, PBMCs are stimulated with antigens for 10–13 days and maintained by fresh media exchange and exogenous IL-2. After this initial culture period, cells are re-stimulated for an additional 20 h in the presence of autologous antigen presenting cells (APC) in anti-IFN-γ coated ELISPOT plates. Studies with samples from humans have demonstrated that cultured ELISPOT responses are primarily a measure of T cell memory (Tcm) cells [27–29].

Sallusto *et al*. [30] identified two functionally distinct subsets of memory T cells (i.e., CD45RA^-^/CD45RO^+^) in mice and humans based on expression of the lymphoid homing receptors CD62L and CCR7. These two subsets are: (1) Tcm cells which express CD62L and CCR7 and are preferentially located in lymphoid tissues and (2) effector memory T (Tem) cells which lack CD62L and CCR7 expression and are preferentially located in peripheral tissues or remain blood associated, either circulating or contained within splenic red pulp or hepatic sinusoids [31]. Tem cells show immediate effector functions, maintaining preformed cytotoxic granules for rapid cytolysis of infected host cells [32]. Tcm cells show elevated proliferation and IL-2 production capabilities, being able to generate Tem and effector cells [33] whereas Tem cells undergo relatively little proliferation and secrete minimal IL-2 upon restimulation [28,31,33]. While Tcm and Tem have different roles in the immune response, both subsets are thought to be important for protection against pathogens. Still, due to the high proliferative capacity and long life span of Tcm, the eliciting of Tcm is believed to provide long-term protection.

The cultured ELISPOT assay measures memory responses, primarily Tcm in humans [27–29]. Godkin *et al*. [27] tracked hepatitis C virus HLA-DR11-restricted epitopes in the course of the long-term culture, demonstrating that cultured ELISPOT IFN-γ production was due to long-lived CD4^+^ Tcm expressing CCR7. The fundamental role of CCR7^+^ CD4 cells was also reported by Todryk *et al*. [34]; these authors assessed the effect of depletion of CCR7 expressing cells on *ex vivo* or cultured ELISPOT responses to either influenza antigenic peptides or *M*. *tb* purified protein derivative (PPD). The depletion of CCR7^+^ cells dramatically reduced cultured ELISPOT responses, yet had only a minimal effect on *ex vivo* responses. Supportive of the idea that the cultured ELISPOT response is a measure of Tcm responses, several studies have shown the association of responses measured by this assay with protection against malaria, suppression of viral recrudescence in hepatitis B virus carriers, low viremia in human immunodeficiency virus (HIV) infection, and favorable outcomes in human TB [28,35–38].

While responses measured by cultured IFN-γ ELISPOT following vaccination correlate to protection with bTB; the phenotype of the responding cells within the long-term cultures has not been determined for cattle in response to neither vaccination nor infection. A better understanding of the cattle immune system may enable the development of improved vaccine strategies and consequently, greater protection against this zoonotic disease of cattle. In the present study, we characterize effector and memory T cell subsets in the immune response to *M*. *bovis* infection of cattle.

## Materials and Methods

### Animal Use Ethics

All studies were approved by the National Animal Disease Center Animal Care and Use (Protocol #’s ACUP-2508 and ACUP-2688) / Institutional Biosafety (Permit #’s IBC-0285A and IBC-0004RA) committees and performed under appropriate project licenses within the conditions of the Animal Welfare Act originally signed into law in 1966 and in accordance with the most recent amendments. All animals were housed in appropriate biological containment facilities at the National Animal Disease Center. Animals did not develop clinical signs of bTB (such as: cough, dyspnea, anorexia and weight loss); however, one animal (from the non-infected control group) was euthanized by intravenous administration of sodium pentobarbital due to an umbilical infection.

### *Mycobacterium bovis* aerosol challenge procedures

Two field strains of *M*. *bovis* were used for challenge inoculum: 95–1315 (Michigan white-tailed deer isolate) and 10–7428 (Colorado dairy isolate). Low passage (≤ 3) cultures of both strains were prepared using standard techniques in Middlebrook 7H9 liquid media (Becton Dickinson, Franklin Lakes, NJ) supplemented with 10% oleic acid-albumin-dextrose complex (OADC) plus 0.05% Tween 80 (Sigma, St. Louis, Missouri). Holstein steers (~ 6 months of age) were obtained from a bTB-free herd in Sioux Center, IA and housed in a biosafety level-3 (BSL-3) facility at NADC in separate rooms based upon treatment group. For the first experiment, treatment groups consisted of non-infected steers (n = 7) and animals receiving 10^4^ colony-forming units (cfu) of *M*. *bovis* 95–1315 (n = 8), or *M*. *bovis* 10–7428 (n = 8). For the second study, a single group of steers (n = 8) received 10^4^ cfu *M*. *bovis* 10–7428. For both studies, *M*. *bovis* challenge inoculum was delivered to restrained calves (~9 months of age) by aerosol as described by Palmer *et al*. [39]. Briefly, inoculum was nebulized into a mask (Trudell Medical International, London, ON, Canada) covering the nostrils and mouth, allowing regular breathing and delivery of the mycobacteria to the lungs via the nostrils. The process continued until the inoculum, a 1 ml PBS wash of the inoculum tube, and an additional 2 ml PBS were delivered—a process taking ~10 min. Strict biosafety protocols were followed to protect personnel from exposure to *M*. *bovis* throughout the study, including BSL-3 containment upon initiation of *M*. *bovis* challenge in animal rooms and standard laboratory practices for handling *M*. *bovis* cultures and samples from *M*. *bovis*-infected animals.

### Mycobacterial isolation and assessment of lesions

All calves were euthanized ~4 months after challenge by intravenous administration of sodium pentobarbital. Tissues were examined for gross lesions and processed for microscopic analysis and isolation of *M*. *bovis*. Tissues collected included: lung; liver; mandibular, parotid, medial retropharyngeal, mediastinal, tracheobronchial, hepatic, and mesenteric lymph nodes. Lymph nodes were sectioned at 0.5 cm intervals and examined. Each lung lobe was sectioned at 0.5–1.0 cm intervals and examined separately. Lungs and lymph nodes (mediastinal and tracheobronchial) were evaluated using a semi-quantitative gross pathology scoring system adapted from Vordermeier *et al*.[9]. Tissues collected for microscopic analysis were fixed by immersion in 10% neutral buffered formalin. For microscopic examination, formalin-fixed tissues were processed by standard paraffin-embedment techniques, cut in 5 μm sections and stained with hematoxylin and eosin. Adjacent sections from samples containing caseonecrotic granulomata suggestive of bTB were stained by the Ziehl-Neelsen technique for identification of acid-fast bacteria. Microscopic tuberculous lesions were staged (I-IV) based on a scoring system developed by Wangoo *et al*. [40].

### *Ex vivo* and long-term cell culture

PBMC were isolated from buffy coat fractions of blood collected in 2 × acid-citrate-dextrose solution. Complete RPMI medium for PBMC cell culture was RPMI 1640 (GIBCO, Grand Island) supplemented with 2 mM L-glutamine, 25 mM HEPES buffer, 100 U/ml penicillin, 0.1 mg/ml streptomycin, 1% non-essential amino acids (Sigma, St. Louis, MO), 2% essential amino acids (Sigma), 1% sodium pyruvate (Sigma), 50 mM 2-mercaptoethanol (Sigma), and 10% (v/v) fetal bovine sera (FBS). Long-term cell cultures were generated by stimulating 2 × 10^6^/ml PBMC with a cocktail of *M*. *bovis* PPD (PPDb, 5 μg/ml, Prionics Ag, Sclieren, Switzerland) rAg85A (1 μg/ml, LIONEX Diagnostics and Therapeutics GmbH, Braunschweig, Germany), rTB10.4 (1 μg/ml, LIONEX Diagnostics and Therapeutics GmbH), and rESAT-6/CFP-10 (1 μg/ml, kind gift from Chris Minion, Iowa State University) in complete RPMI medium. Cells were cultured (2 × 10^6^ cells/well, 1 ml/well) in 24 well flat-bottom microtiter plates (Nunc, Thermo Fisher, Waltham, MA). Media containing human rIL-2 (Sigma, 10 U/ml) was used to replace media from the PBMC cultures at days 3 and 7. Fresh media without IL-2 was used at days 10 and 12. For ELISPOT assays, at day 13, cultured cells were added (2 × 10^4^ of cultured PBMC/well) to anti-bovine IFN-γ capture-mAb (Serotec, Oxford, UK) coated 96-well ELISPOT plates (Millipore, Watford, UK) and incubated in the presence of autologous APCs and either PPDb (5 μg/ml), rESAT-6:CFP10 (1 μg/ml), pokeweed mitogen (PWM, Sigma) (1μg/ml) or medium alone. Autologous APCs were isolated by adherence incubating 1 × 10^5^ freshly isolated PBMC in complete medium at 39°C/5% CO_2_ for 90 min in ELISPOT plates. Non-adherent cells were discarded and the adherent cells (APCs) washed four times with warm RPMI 1640 media. Fresh complete media containing antigen and long-term cultured cells were then incubated 20h at 39°C/5% CO_2_. For flow cytometric analysis, cultured cells were added (2 × 10^4^ of cultured PBMC/well) to round-bottom well plates and incubated in the presence of APCs and either PPDb (5 μg/ml), rESAT-6:CFP10 (1 μg/ml), PWM (1 μg/ml) or medium alone. Autologous APCs were prepared as described for the ELISPOT assay. Fresh complete media containing antigen and long-term cultured cells were then incubated 16 h at 39°C/5% CO_2_ with Brefeldin A (Sigma, 10 μg/ml) added at 4 h of culture.

### IFN-γ ELISPOT

The IFN-γ ELISPOT assay was performed as described by Vordermeier *et al*. [41] and Whelan *et al*., [25]. Briefly, polyvinyldifluoride 96-well ELISPOT plates (Millipore) were coated at 4°C overnight with an anti-bovine IFN-γ capture mAb (Serotec), followed by a blocking step (10% fetal bovine serum (FBS) in RPMI 1640 media, for 2 h at 39°C/5% CO_2_). Autologous APCs were isolated by adherence on coated and blocked ELISPOT plates. Long-term cultured cells (2 × 10^4^ of cultured PBMC/well) were added to ELISPOT plates (Millipore, Watford, UK) and incubated with either PPDb (5 μg/ml), rESAT-6:CFP10 (1 μg/ml), PWM (1 μg/ml) or medium alone for 20 h at 39°C/5% CO_2_. Spot forming cells (SFC) were detected following the Vectastain ABC-AP Kit (Vector Laboratories, Burlingame, CA) standard procedures.

### Flow Cytometry

Following the appropriate culture duration, cells were pooled from individual animals according to *in vitro* treatments (i.e., stimulation). Cells were stained as described by Maue *et al*. [42], with the primary antibodies and appropriate secondary antibodies listed on the Table 1. CD4 T cells were analyzed as a separate panel, while CD8 and γδ T cells staining was performed together to enable the analysis of CD8 expressing γδ T cells. Intracellular staining was performed following BD Perm/Wash instructions (BD Biosciences, San Jose, CA).

**Table 1:** Primary and secondary monoclonal antibodies and proliferation staining reagents.

Reagent or antibody	Specificity, Source	Secondary antibodies, Source
**ILA11**	Bovine CD4, Washington State University	Alexa-fluor 350, Life Technologies
**ILA116**	Bovine CD45RO, Washington State University	FITC, SouthernBiotech or Allophycocyanin-Cy7, Life Technologies
**7D12**	Human CCR7, BD Pharmingen	Allophycocyanin, SouthernBiotech
**BAT31A**	Bovine CD44, Washington State University	PE-Cy7, SouthernBiotech or Pacific blue, Life Technologies
**BAQ92A**	Bovine CD62L, Washington State University	Percp, SouthernBiotech
**MCA1783-PE**	Bovine IFN-γ, AbD Serotec	Not applicable
**GB21A**	Bovine TCR1 δ chain, Washington State University	PE-Cy7, SouthernBiotech
**BAQ111A**	Bovine CD8, Washington State University	PE, SouthernBiotech
**CellTrace Violet**	Not applicable, Life Technologies	Not applicable

For cell-trace labeling, cells were labeled with CellTrace Violet (Invitrogen, Carlsbad, CA) following kit instructions. Briefly, either freshly isolated or long-term cells (cultured for 13 days) were resuspended at 1 × 10^7^ cells in PBS containing 10 μM/ml of the cellTrace dye with immediate vortexing to ensure rapid homogenous staining of cells. Staining was performed at 20°C and cell were incubated for 5 min. Cells were washed three times with PBS containing 10% FBS and cultured for additional six days in the presence of APCs and antigens in round-bottom 96-well plates before cell staining with primary and secondary antibodies. For the long-term cultured cells the culture length was 19 days. Flow cytometric analysis was performed with a BD LSR flow cytometer (BD Biosciences). Data were analyzed using FlowJo (Tree Star Inc., San Carlos, CA).

### Statistical analysis

Data were analyzed using Analysis of Variance followed by Tukey’s or Šídák’s multiple comparisons test or Student’s *t* test using GraphPAD Prism 6.0 (GraphPAD Software Inc., La Jolla, CA).

## Results

### Aerosol *M*. *bovis* infection of cattle elicits long-term cultured IFN-γ ELISPOT responses

Aerosol inoculation to cattle with *M*. *bovis* 95–1315 or *M*. *bovis* 10–7428 resulted in a similar distribution and severity of gross and microscopic tuberculous lesions as well as mycobacterial colonization, primarily affecting the lungs and lung-associated lymph nodes [43]. Specific cell-mediated and antibody responses, including kinetics of the response as well as antigen recognition profiles, were also comparable between the two treatment groups [43,44].

**Fig 1:**
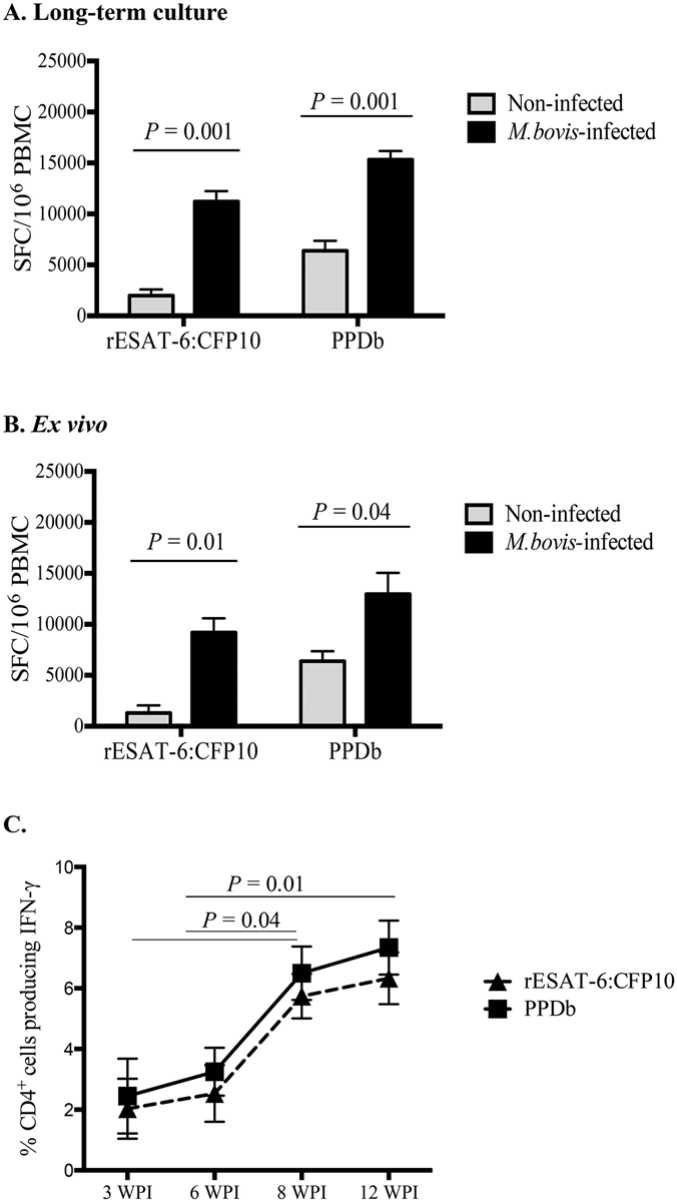
Long-term cultured and *ex vivo* IFN- γ responses by cattle after *M*. *bovis* aerosol challenge. Cultured ELISPOT analysis was performed ~3 weeks after challenge with virulent *M*. *bovis*. Long-term cultured cells were generated by stimulating PBMC with a cocktail of rAg85A (1 μg/ml), rTB10.4 (1 μg/ml), and rESAT-6:CFP10 (1 μg/ml) antigens as well as PPDb (5 μg/ml) for 13 days followed by transfer to ELISPOT plates with APCs and addition of either rESAT-6:CFP10, PPDb or medium alone. For the *ex vivo* response, freshly isolated PBMCs were stimulated with rESAT-6:CFP10, PPDb or medium alone for 16h. Medium control responses were subtracted from antigen-stimulated responses and results are presented as mean spot forming cells (SFC)/million cells (± SEM, n = 8) for **(A)** long-term culture or **(B)** *ex vivo* conditions. **(C)** The kinetics of the response is shown as the percent of CD4^+^ cells producing IFN-γ in long-term cultures at 3, 6, 8, and 12 weeks post infection (WPI n = 6). Two-way ANOVA (Šídák’s multiple comparison post-test).

With bTB vaccine efficacy studies, long-term cultured (i.e., 14 days) IFN-γ ELISPOT responses to vaccination (i.e., BCG, *M*. *bovis* ΔRD1, and viral-vectored Ag85) negatively correlates with mycobacterial burden and TB-associated pathology and positively correlates with vaccine-induced protection [23–25]. As with vaccination, *M*. *bovis* infection also elicited long-term cultured IFN-γ ELISPOT responses in cattle (Fig 1). Three weeks after infection, long-term cultured IFN-γ ELISPOT responses by PBMCs from infected cattle to rESAT-6:CFP10 and PPDb exceeded (*P < 0*.*05*) respective responses by PBMCs from non-infected calves (Fig 1A). Similar results were detected with *ex vivo* (i.e., short-term) responses (Fig 1B). Also, Tcm and *ex vivo* responses did not differ (*P > 0*.*05)* between *M*. *bovis* 95-1315- and 10-7428-infected groups (S1 Fig). The weak response detected to PPDb by non-infected cattle in both *ex vivo* and long-term cultures was likely due to prior exposure to non-tuberculous mycobacteria (NTM, a common occurrence in US dairy cattle) as pre-infection whole blood (18 h stimulation) IFN-γ responses to *M*. *avium* PPD (PPDa) exceeded (*P < 0*.*05*) respective responses to PPDb (S2 Fig). Using intracellular cytokine staining, IFN-γ responses to PPDb and to rESAT-6:CFP10 were also detected in long-term PBMC cultures at 3, 6, 8, and 12 weeks after aerosol infection (Fig 1C). Responses increased (*P < 0*.*05)* from 3 and 6 to 12 weeks after infection. These findings demonstrate that infection of cattle with virulent *M*. *bovis* elicits long-term cultured IFN-γ ELISPOT responses, which are considered a surrogate of Tcm responses in humans [34,38,45].

### Analysis of IFN-γ production in long-term cultures reveals a dominant contribution by Tcm

**Fig 2:**
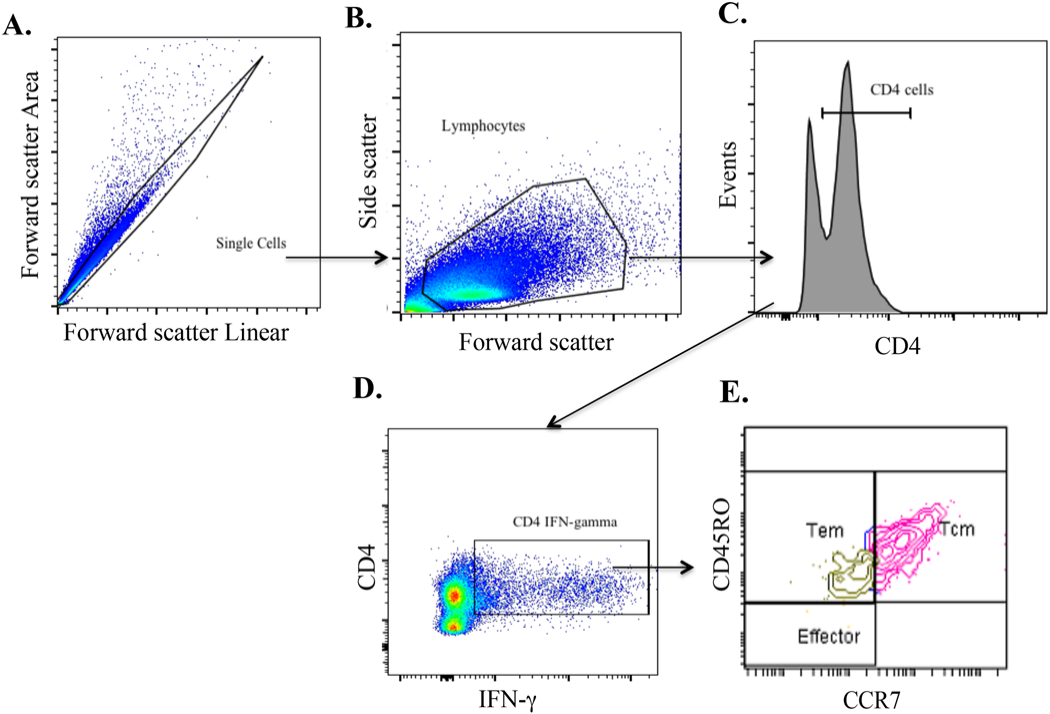
Representative gating strategy for evaluation of CD45RO and CCR7 expression on CD4 T cells producing IFN-γ. Approximately 8 weeks after aerosol challenge with *M*. *bovis*, long-term cultures were generated by stimulating PBMC with a cocktail of rAg85A (1 μg/ml), rTB10.4 (1 μg/ml) and rESAT-6:CFP10 (1 μg/ml) as well as PPDb (5 μg/ml) for 13 days followed by transfer of cells to ELISPOT plates with APCs and restimulation with PPDb. Gating hierarchy (gating sequence as depicted by the arrows): **(A)** Single cells (within the oblong gate), **(B)** Lymphocytes (within the polygon gate), **(C)** CD4^+^ cells, **(D)** CD4^+^ cells producing IFN-γ, and **(E)** CD45RO and CCR7 expression for determination of effector/memory phenotypes.

**Fig 3:**
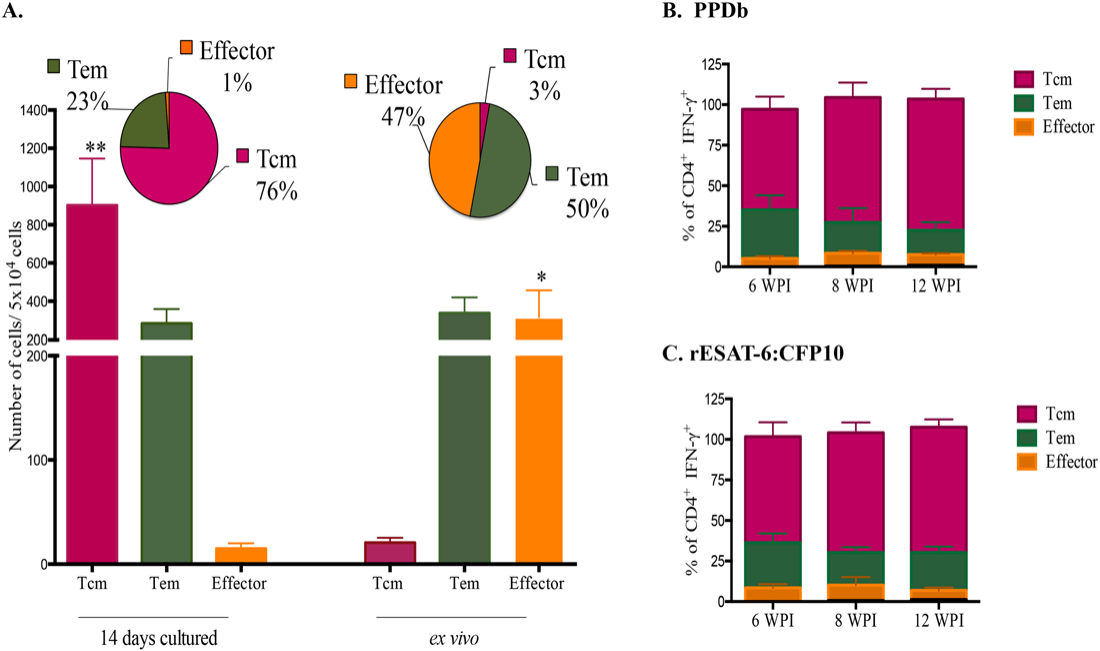
Frequencies of Tcm, Tem and effector cells producing IFN-γ in response to mycobacterial antigens in long-term and *ex vivo* assays. Peripheral blood mononuclear cells were isolated from calves ~ 8 weeks after challenge with virulent *M*. *bovis*. Cells were stimulated with a cocktail of rAg85A (1 μg/ml), rTB10.4 (1 μg/ml), and rESAT-6:CFP10 (1 μg/ml) as well as PPDb (5 μg/ml) for 13 days followed by transfer to 96 well round bottom plates with APCs and addition of media alone, PPDb or rESAT-6:CFP10 for an additional 16h. For *ex vivo* culture, PBMC were stimulated with media alone, PPDb or rESAT-6:CFP10 for 16 h. **(A)** Relative contribution of Tcm, Tem, and T effector cells to IFN-γ production in response to PPDb by long-term (i.e., 14-day) (left) and *ex vivo* (i.e., 16 h) (right) cultures, 8 weeks after *M*. *bovis* challenge. Data are presented in percentages (pies) and as mean (± SEM) number of cells producing IFN-γ **/** 10^4^ cells (histograms) (n = 16). Relative contribution of Tcm, Tem and T effector cells to IFN-γ production in response to PPDb **(B)** or to rESAT-6:CFP10 **(C)** in long-term cultures at three, six, eight or 12 weeks post-infection (WPI, n = 6). Tcm, Tem and effector cell phenotypes were as defined in Fig 2 and S3 Fig Tcm and Effector T cell contribution to IFN-γ production differs (*P < 0.05; **P < 0.01, paired Student's t-tests) between short- and long-term cultures.

The expression of CD45RO, CD4, CCR7 and intracellular expression of IFN-γ by PBMC cells was evaluated following long-term or *ex vivo* culture (Fig 2). CD4 T cells producing IFN-γ following long-term culture predominantly co-expressed CD45RO and CCR7 surface antigens (Fig 3), consistent with the Tcm phenotype described for humans and mice (S3 Fig) [26],[46]. The phenotype of cells responding to PPDb (CD4^+^ IFN-γ^+^) was compared under *ex vivo* versus long-term culture conditions (Fig 3A and S4A Fig). The predominant cell phenotype responding to antigenic stimulation in long-term cultures was that of Tcm cells, whereas few Tcm were present under *ex vivo* conditions (P < 0.01, % Tcm in long-term versus *ex vivo* cultures). In contrast, effector cells contributed to *ex vivo* IFN-γ production, but only minimally to the long-term culture response (P < 0.05). Tem cells contributed to IFN-γ production in both *ex vivo* (~50%) and long-term cultures (~25%). The respective overall effector/memory CD4 T cells (i.e. CD4^+^ IFN-γ^+/-^ cells) proportions under both long- and short-term conditions are shown in S4B Fig The relative contribution of Tcm, Tem and effector CD4^+^ T cells in the response to PPDb (Fig 3B) and to rESAT-6:CFP10 (Fig 3C) remained the same over the course of infection (i.e., at 6, 8, and 12 weeks after challenge). In general a greater number of responding cells (IFN-γ^+^) were observed in the long-term cultured assay as compared to the *ex vivo* assay (Fig 3), perhaps due to the greater percentage of CD4 cells within long-term cultures (S5 Fig).

**Fig 4:**
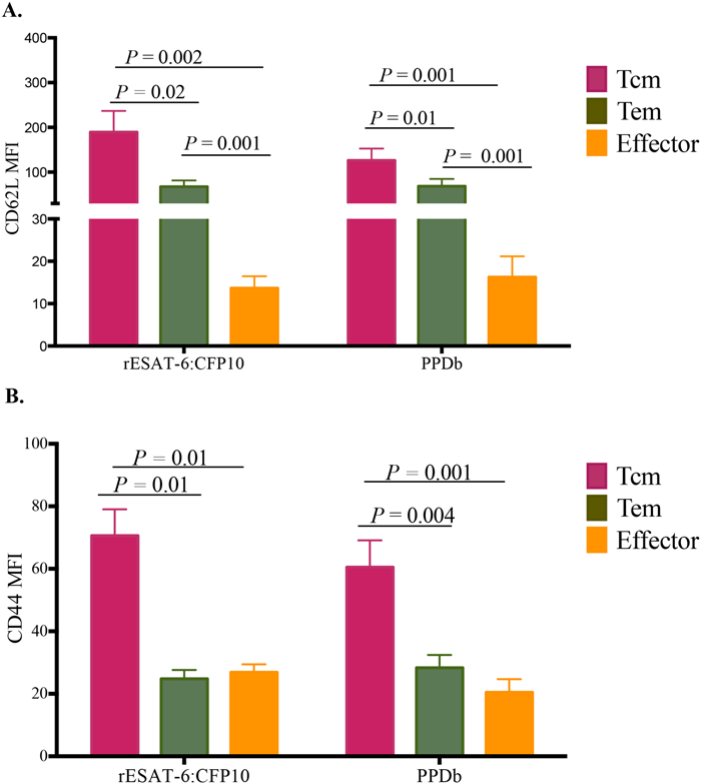
CD62L and CD44 expression by Tcm, Tem and effector CD4+ cells in long-term (14 day) cultures. Analysis of long-term cultured PBMCs was performed ~ 8 weeks after aerosol challenge with virulent *M*. *bovis*. Cells were stimulated with a cocktail of rAg85A (1 μg/ml), rTB10.4 (1 μg/ml), and rESAT-6:CFP10 (1 μg/ml), as well as PPDb (5 μg/ml) for 13 days followed by transfer to 96 well round bottom plates with APCs and addition of PPDb or rESAT-6:CFP10. Data are presented as mean fluorescence intensity (MFI, y-axis, ± SEM) of CD62L or CD44 by CD4^+^ cells of the various effector / memory subsets (x-axis). **(A)** CD62L expression by Tcm, Tem and effector cells in response to PPDb and rESAT-6:CFP10. **(B)** CD44 expression on Tcm, Tem and effector cells in response to PPDb and rESAT-6:CFP10. Tcm, Tem and effector cell phenotypes were as defined in Fig 2 and S3 Fig Paired Student's t-tests (n = 8).

Tcm cells highly expressed (P < 0.05) CD62L and CD44 in response to either rESAT-6:CFP10 or PPDb stimulation (Fig 4). Expression of CD62L was intermediate with Tem and low to non-existent with effector cells (Fig 4A). CD44 expression was low in both Tem and effector cells (Fig 4B).

### Tcm cells possess high proliferative capability

**Fig 5:**
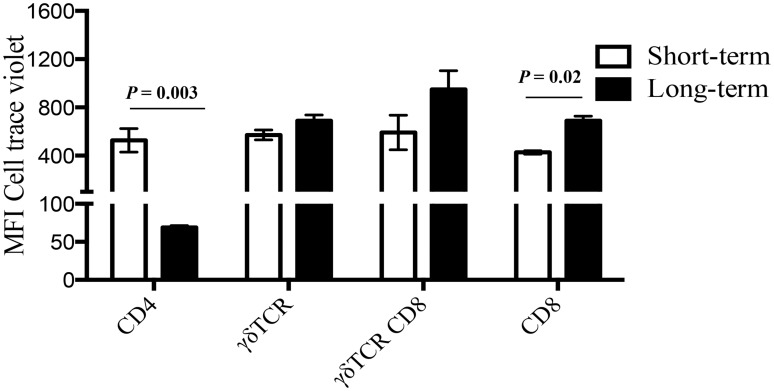
Proliferation of cell subsets in response to mycobacterial antigens. Approximately 7 weeks after *M*. *bovis* challenge, PBMCs from infected cattle were either long or short-term cultured (6 days). Long-term cells consisted of PBMCs cultured in the presence of rAg85A, rTB10.4, rESAT-6:CFP10 and PPDb for 13 days (n = 4). Subsequently, cells were stained with CellTrace violet dye and re-stimulated with either rESAT-6:CFP10 or medium in the presence of APCs for an additional six days. Short-term cells consist of CellTrace Violet-stained PBMC from *M*. *bovis*-infected cattle cultured for six days in the presence of either rESAT-6:CFP10 or medium (n = 4). Proliferative responses of cells under both culture conditions were compared for each T cell subset. Data are presented as mean fluorescence intensity (MFI, y-axis) of CellTrace Violet for analysis of proliferation of T cell subsets (x-axis). Two-way ANOVA, n = 6, Šídák’s multiple comparison post-test.

**Fig 6:**
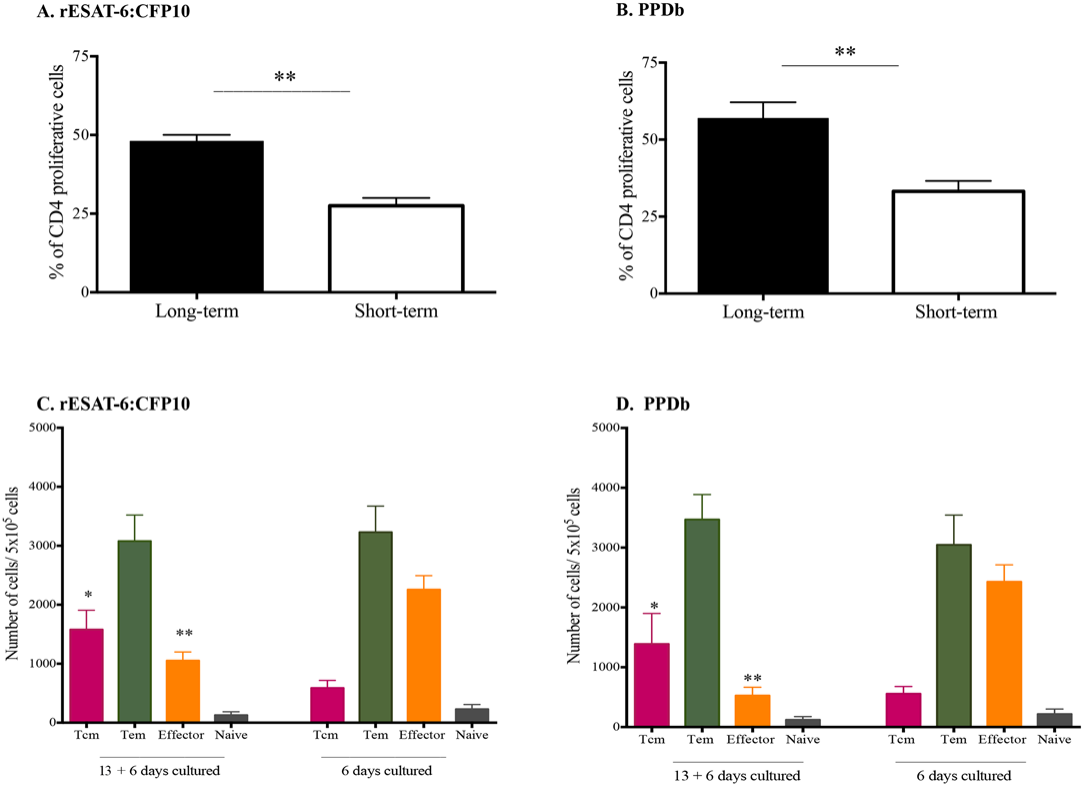
Long-term cultured cells have higher proliferative responses than short-term cells. Long-term and short-term cultured PBMCs were analyzed ~ 7 weeks after aerosol challenge with virulent *M*. *bovis*. Long-term cells consist of PBMC from *M*. *bovis* aerosol infected cattle cultured in the presence of rAg85A, rTB10.4, rESAT-6:CFP10 and PPDb for 13 days and then CellTrace violet-stained and re-stimulated with either rESAT-6:CFP10, PPDb or medium in the presence of fresh autologous adherent cells for an additional six days. Short-term cells consist of CellTrace violet-stained PBMC from *M*. *bovis* aerosol infected cattle cultured for six days in the presence of either rESAT-6:CFP10, PPDb or medium. The gating strategy was performed in accordance with procedures described in Fig 2A for single cells, and as shown in S3 Fig for lymphocytes, and CD4^+^ cells. **(A)** Percentages of CD4^+^ cells proliferating (low Celltrace dye MFI) in response to rESAT-6:CFP10 within long or short-term cultures. **(B)** Percentages of CD4^+^ cells proliferating (low Celltrace dye MFI) in response to PPDb within long or short-term cultures. **(C)** Memory/effector phenotype of proliferating CD4^+^ cells within long-term or short-term cultures in response to rESAT-6:CFP10. **(D)** Memory/effector phenotype of proliferating CD4^+^ cells within long-term or short-term cultures in response to PPDb. For panels A and B, cell proliferation differs (**P < 0.01, n = 6 paired Student's t-tests) between long and short-term cultures to either rESAT-6:CFP10 or PPDb. For panels C and D, Tcm and effector cell content differs (*P < 0.05; **P < 0.01, n = 4 paired Student's t-tests) between short-term and long-term cultures to either rESAT-6:CFP10 or PPDb.

Human CD4 memory T cells, predominantly those exhibiting Tcm phenotype, proliferate in response to cytokine and antigenic stimulations, differentiating into Tem or effector T cells *in vitro* [30,47]. To assess the proliferative capacity of bovine Tcm cells following long-term culture, cells were harvested at day 13 and stained with CellTrace Violet. CellTrace Violet stained cells were re-stimulated with rESAT-6:CFP10 or PPDb for additional six days, without IL-2 (S5 Fig). For comparative purposes, freshly isolated PBMC were isolated and stained with CellTrace Violet and cultured for six days (short-term culture). Cells proliferated in response to antigenic stimulation under both long- and short-term conditions (Table 2). CD4 T cells were the most proliferative fraction (*P < 0*.*05*), followed by **γ**δ, CD8 T cells, and CD8 expressing γδ T cells. In response to rESAT-6:CFP10 stimulation, the number of CD4 T cells proliferating in long-term cultures exceeded (*P < 0*.*05*) that of short-term cultures. Similarly, the CellTrace Violet mean fluorescence intensity (MFI) was significantly lower (*P = 0*.*003*), indicating greater cell proliferation in rESAT-6:CFP10-stimulated CD4 T cells in long- vs short-term cultures (Fig 5 and Fig 6). Greater percentages of CD4 T cells (*P < 0*.*05*) proliferated under long-term culture in response to either rESAT-6:CFP10 (Fig 6A) or PPDb (Fig 6B). These findings demonstrate that bovine Tcm cells are highly proliferative in response to repeated stimulation with recall antigen.

**Table 2:** Lymphocyte subset proliferative responses of *M*. *bovis*-infected cattle under short or long-term culture condition.

Culture	Stimulation	Mean cell number (± SEM) by cell subset (50,000 cells)				
		Ungated	CD4^+^	CD8^+^	γδ TCR^+^	γδ TCR^+^ CD8^+^
**Long-term culture**	rESAT-6:CFP-10	10,937 (±1476)	8,915 (±1,231) *	113 (±28) *	996 (±382)	51 (±9)
**Short-term culture**	rESAT-6:CFP-10	7,407 (±975)	4,076 (±567)	468 (±197)	942 (±17)	141 (±27)
**Long-term culture**	*M*. *bovis* PPD	15,061(±2187)	6,107 (±1343)	159 (±45) *	1354 (±68)	64 (±19)
**Short-term culture**	*M*. *bovis* PPD	13,146 (±2835)	4,841 (±1742)	496 (±12)	1612 (±427)	89 (±20)

Long-term cells consist of PBMC from *M*. *bovis*-infected cattle cultured in the presence of rAg85A, rTB10.4, rESAT-6:CFP10 and PPDb for 13 days. Subsequently, cells were stained with CellTrace violet dye and re-stimulated with either, PPDb, rESAT-6:CFP10 or medium in the presence of APCs for an additional six days. Short-term cells consist of CellTrace Violet-stained PBMC from *M*. *bovis*-infected cattle (n = 4) cultured for six days in the presence of either PPDb, rESAT-6:CFP10 or medium in the presence of APCs. Data are presented as the mean (±SEM) number of cells that had proliferated per 50,000 cells under short-term or long-term culture in response to antigen (PPDb or rESAT-6:CFP10) minus the response to media alone. *Responses under long-term culture differ (*P* < 0.05,two-way ANOVA, Šídák multiple comparison post-test) from the respective short-term culture (i.e., relative to lymphocyte subset and antigen stimulation).

Given the robust participation of CD4 T cells in the long-term proliferative response and the fact that Tcm cells are believed to generate effector cells, the phenotype of these cells (i.e., 14-day cultured cells re-stimulated with antigen for an additional 6 days) was further assessed. Again, PBMCs cultured under short-term culture conditions (6 days only) were used for comparison (Fig 6). The distribution of CD4 T cells into Tcm and effector subsets was different between short- and long-term conditions following stimulation with rESAT-6:CFP10 (Fig 6C) or PPDb (Fig 6D). Greater (*P < 0*.*05*) numbers of Tcm cells were present in long-term (13 + 6 days) versus short-term (6 days) cultures; while more effector cells were present (*P < 0*.*05*) in short—versus long-term cultures. The numbers of Tem and naïve cells were similar between the two conditions. Tem cells were the main population in both cultures, whereas few naïve cells persisted under either culture condition (Fig 6C and 6D). These findings indicate that highly proliferative Tcm cells partially revert to effector (predominately Tem) phenotypes upon additional exposure to antigen for 6 days (Fig 6C and 6D as compared to Fig 3).

## Discussion

This study characterizes Tcm cells in cattle and their participation in the immune response to *M*. *bovis* infection. As early as 6 weeks after *M*. *bovis* infection, CD4^+^ Tcm cells (CD45RO^+^, CCR7^+^) were detected in long-term, antigen-stimulated PBMC cultures upon recall stimulation with specific (i.e., rESAT-6:CFP-10) or complex (i.e., PPDb) antigens of *M*. *bovis* (Fig 3). Antigen-specific CD4 cells, as detected by IFN-γ production via either ELISPOT (Fig 1A and 1B) or intracellular cytokine staining (Figs 1C, 2 and 3), within long-term PBMC cultures were predominately (~76%) Tcm cells (CD45RO^+^ / CCR7^+^), with the remainder being Tem cells (CD45RO^+^ / CCR7^-^, ~23%) (Fig 3). Bovine Tcm were highly proliferative as antigen restimulation of cells within long-term cultures induced robust proliferation of CD4^+^ cells that significantly exceeded that of short-term culture cells (Table 2, Figs 5, 6A and S5 Fig). Further phenotypic analyses of repeat stimulated cultures indicated that a sub-population of bovine Tcm reverted to effector (both Tem and T effector) phenotypes upon repeat exposure to *M*. *bovis* antigens (Fig 6B). The identification and characterization of CD4 Tcm and Tem subpopulations in cattle should prove useful for development of vaccines and the understanding of the immunopathogenesis for many infectious diseases of cattle.

Memory cells elicited either by vaccination [30,48] or during pathogen clearance [15,32] are thought to provide long-term protection due to their prolonged life-span, proliferation potential, and plasticity [7,33,48]. While events governing immunological memory during chronic infections (wherein the antigenic stimulation persists) are not well understood, a significant Tcm response is associated with a favorable outcome for chronic infections, such as HIV and TB (e.g., latency and self-healing with TB and subclinical disease with HIV) [31,38]. Still, the relative importance of Tcm for protective immunity against TB is not fully established. Tcm and *ex vivo* responses are detected in *M*. *tb*-infected patients [33,37] and loss of Tcm responses (as measured by cultured IFN-γ ELISPOT) is associated with clinical disease progression [27,37]. Likewise, Tcm responses in the absence of *ex vivo* IFN-γ production indicate disease remission, either by self-healing [34,38] or anti-mycobacterial therapy, reinforcing the role that pathogen clearance has on Tcm function and/or maintenance [34,37]. Intriguingly, in spite of the presence of Tcm cells, patients receiving curative treatment are still susceptible to *M*. *tb* re-infection [28,31,49]. In the current set of experiments, each of the calves had mild progressive disease and were responsive to TB antigens in both cultured and *ex vivo* IFN-γ ELISPOT assays—similar to what occurs in humans with the mild active form of *M*. *tb* infection [38]. Flow cytometric analysis demonstrated that both Tem and Tcm cells were elicited relatively early after infection (3 weeks post-infection, Fig 1). It is uncertain if Tcm responses by the animals would decrease as the disease progresses, but it is frequently reported that animals in late stages of infection become anergic to measures of cell-mediated immunity, yielding false negative results upon skin test or *ex vivo* IFN-γ assays [50–52]. Prolonged infection trials with cattle are difficult due to biocontainment costs and ethical issues associated with extended duration of housing large animals within restrictive facilities. Thus, further studies to characterize the progression of Tcm responses to *M*. *bovis* infection in cattle may require sampling of field reactors with various clinical manifestations of the disease including: chronic progressive disease, *M*. *bovis* detected yet no visible lesions (i.e., analogous to latency in humans), and severe disseminated disease.

Although IFN-γ is key to a successful containment of mycobacterial infections, the interaction between pathogens and host is intricate. Several factors and cytokines may be relevant to *M*. *bovis* infection outcome. Together with IFN-γ, tumor necrosis factor alpha (TNF-α) is crucial for the control of mycobacterial infections of humans and mice [53]. Also, T cells producing multiple cytokines have been recently identified and may play an important role in infection outcome. Polyfunctional T cells co-producing IFN-γ, TNF-α and IL-2 are associated with infection control in HIV, hepatitis C, leishmaniasis and malaria (reviewed in [54,55]). With TB, conflicting data indicate that polyfunctional T cell responses are associated with either clinical disease (i.e., as a biomarker of active TB) or infection control [55]. Polyfunctional T cells were also recently described in cattle in the response to *M*. *bovis* infection [56] and to anti-mycobacterial vaccines [57]. Studies on polyfunctional responses of bovine memory T cells under our system (long-term cultured cells) are currently in progress. Additionally, TNF-α and IL-2 have been evaluated in cattle for diagnostic purposes [58,59].

Upon *M*. *tb* infection, the primary immune response may take days to weeks to develop relying on exposure of naive T cells to antigens in secondary lymphoid organs, expansion of antigen-specific cells, and homing of effector cells to the site of infection [60]. With TB, this 2–3 week delay in the response at the primary site of infection seems to be advantageous for the bacteria, and is observed during infection in cattle, humans, and mice [61]. In theory, the delay allows the pathogen an opportunity to establish a niche and to direct the ensuing immune response in favor of bacterial persistence and chronic immune stimulation. A more rapid homing of antigen-specific cells to the site of infection (e.g., as may occur in vaccinated animals) might be advantageous to the host, allowing more effective induction of immune responses (e.g., different activation status, cytokine profile) and circumvention of the pathogens regulation of host immunity [14,62,63]. These factors may partially explain why Tcm responses elicited by vaccination appear to be beneficial to the host, while similar Tcm responses are detected in infected animals with progressive disease. The presence of a robust TB-specific Tcm population elicited by vaccination prior to infection may lead to pathogen clearance before establishment of immune evasion tactics by the pathogen. Present findings provide a basis for future studies to determine the relative role of these Tcm and Tem subsets in vaccine-elicited protection.

Lymphocyte homing and trafficking to sites of inflammation and lymphoid organs are mediated by the expression of numerous surface adhesion molecules, such as CCR7, CD62L and CD44. CD62L mediates cell adhesion to peripheral lymph node vascular addressins (e.g., GlyCAM-1 and MAdCAM-1) [64]. The expression of CD62L on naïve and memory T cells facilitates cell rolling on endothelium in secondary lymphatic organs, contributing to the compartmentalization of the immune response [64]. CD44 expression on T cells is up regulated upon activation, thereby promoting movement through the extracellular matrix via interactions with hyaluronic acid and fibronectin [65]. *In vitro* stimulation of antigen specific T cells is known to up-regulate CD44 while concurrently down regulating CD62L expression in humans [66], mice [67] and cattle [56,68]. As in humans [66], bovine T cells expressing CD44 upon *ex vivo* stimulation frequently co-express CD45RO, while down regulating CD62L [56,68]. In the present study, CD44 expression on CD4 cells did not differ between Tem and effector cells under *ex vivo* or long-term culture conditions (Fig 4B). It is noteworthy that in the present study the expression of CD62L and CD44 was evaluated only among IFN-γ producing cells; thus, comparisons between resting/unstimulated versus responding/stimulated cells were not performed as previously described [68]. Also, the gating strategy employed (based on CCR7 and CD45RO expression) may also have contributed to the apparently discrepant findings. In the present work, the expression of CD62L was down regulated in effector cells, intermediate on Tem cells, and high on Tcm cells (Fig 4A). Although Tcm cells highly expressed CD62L, these cells maintained high CD44 expression. The relevance of the level of CD44 expression by memory T cells is controversial for both mice [15,69,70] and humans [66,69]. Although few authors investigated T cell memory responses in cattle [24,26,41,71–73], to our knowledge no other description of the expression of CD44 by bovine Tcm cells (as defined by CCR7 and CD45RO expression) has been published, making direct comparisons not possible. However, data from mice suggest that although the expression of CD44 is dispensable for early expansion, trafficking and cytokine production of Th1 cells; expression of CD44 is required for long-term cell survival and anamnestic responses to re-infection [70]. In humans and mice it is long known that, together with CD62L, CCR7 plays a major role for cell homing to secondary lymphoid organs (SLO). For cattle, CCR7 expression is required for CD4 T cell migration to SLO, while homing of **γ**δ T cells to SLO is not mediated by CCR7 expression [74]. To our knowledge further cytometric analysis of CCR7 positive cells into Tcm and naïve subsets, as well the antigen specific memory response to infection has not been done in cattle. Totté *et al*. [73] reported a subset of CD4 cells with Tcm characteristics after *Mycoplasma mycoides* infection and pathogen clearance. Upon restimulation, cells expressing CD62L were highly proliferative, while being less prone to down regulate CCR7 transcription. Conversely, cells lacking CD62L showed greater IFN-γ production, lower proliferation and down regulation of CCR7 transcription. Our findings indicate that in response to antigenic stimulation, Tcm cells strongly proliferated (Figs 5 and 6A) and were capable of switching to Tem and effector cells (CCR7 down regulation—Fig 6B).

We also analyzed the contribution of **γ**δ, CD8 and CD8-expressing **γ**δ T cells to the proliferative response of long- and short-term cultures. In cattle, CD8^+^ T-cells can bear either α/β or γ/δ TCR, with profound impacts on antigen specificity and immunological memory, as classical antigen-specific immunological memory resides in α/β T-cells [75,76]. In BCG vaccinated animals, CD8 memory responses were elicited within the CD8 α/β TCR expressing cells, but not the CD8 expressing γδ T cells [76]. In the present study, **γ**δ CD8^+^ T cells showed greater proliferation under short-term culture conditions, while the proliferative response of **γ**δ^+^ CD8^+^ T cells did not differ based on culture length (i.e., short- vs long-term). However, γδ^+^ CD8^+^ T cells constituted only a small population under both culture conditions (Table 1).

In summary, the present study demonstrates Tcm cells in the response to experimental *M*. *bovis* challenge. Our findings suggest that upon repeat *in vitro* stimulation Tcm cells proliferate into different effector cell types. The association of Tcm cells with vaccine-elicited protection, as well as in infection outcome, is still to be determined.

## Supporting Information

S1 Fig Cattle infected with either *M*. *bovis* 95–1315 or *M*. *bovis* 10–7428 have similar long-term cultured and *ex vivo* IFN-γ responses after *M*. *bovis* aerosol challenge. Cultured ELISPOT analysis was performed ~3 weeks after challenge with virulent *M*. *bovis*. Long-term cultured cells were generated by stimulating PBMC with a cocktail of rAg85A (1 μg/ml), rTB10.4 (1 μg/ml), and rESAT-6:CFP10 (1 μg/ml) antigens as well as PPDb (5 μg/ml) for 13 days followed by transfer to ELISPOT plates with APCs and addition of either rESAT-6:CFP10, PPDb or medium alone. For the *ex vivo* response freshly isolated PBMCs were stimulated with rESAT-6:CFP10, PPDb or medium alone for 16h. Medium control responses were subtracted from antigen-stimulated responses and results are presented as mean spot forming cells (SFC)/million cells (± SEM, n = 8) for **(A)** long-term culture or **(B)** *ex vivo* conditions. Responses did not differ between *M*. *bovis* 95–1315 and *M*. *bovis* 10–7428 infection groups (Two-way ANOVA, followed by Tukey’s multiple comparison).
(TIF)

S2 Fig Evaluation of IFN-γ responses to *M*. *avium*-derived PPD (PPDa) and *M*. *bovis*-derived PPD (PPDb) before challenge. Responses to PPDa and PPDb prior to challenge were examined using a commercial IFN-γ release assay (i.e., Bovigam, Prionics Ag, Schlieren, Switzerland) according to manufacturer instructions. Briefly, duplicate 250l heparinised whole blood aliquots were distributed in 96-well plates with PPDb (10g/ml, Prionics Ag), PPDa (10g/ml, Prionics Ag), or no antigen and incubated at 39°C/5% CO_2_ for 20 hours. IFN-γ concentrations in stimulated plasma were determined using a commercial ELISA-based kit (Bovigam, Prionics Ag). Absorbencies of standards (recombinant bovine IFN-γ; Endogen, Rockford, IL) and test samples were read at 450 nm using an ELISA plate reader (Molecular Devices, Menlo Park, CA). Duplicate samples for individual treatments were analyzed and data presented as optical densities at 450 nm of the response to PPDb or PPDa minus the response to no-antigen (mean ± SEM). *Response to PPDa exceeded (*P* < 0.05, n = 24, paired Student's t-test) the response to PPDb.
(TIF)

S3 Fig Cell function and expression of cell markers on different human T cell memory subsets. **(A)** Identification of memory subsets in the human peripheral blood based on the expression of CD45R0, CCR7, CD62L. **(B)** The differentiation of T cells occurs simultaneously with changes in cell functions. Adapted from Mahnkea *et al*. [46]
(TIF)

S4 Fig Effector/memory phenotype of cells producing IFN-γ in response to PPDb under long-term and *ex vivo* conditions by infection group (A) and the respective efector/memory distribution of total CD4 cells, both IFN-γ^+^ positive and IFN-γ^-^ (B). Peripheral blood mononuclear cells were isolated from calves ~ 8 weeks after challenge with virulent *M*. *bovis* (n = 8). Cells were stimulated with a cocktail of rAg85A (1 μg/ml), rTB10.4 (1 μg/ml), and rESAT-6:CFP10 (1 μg/ml) as well as PPDb (5 μg/ml) for 13 days followed by transfer to 96 well round bottom plates with APCs and addition of media alone, PPDb or rESAT-6:CFP10 for an additional 16h. For *ex vivo* culture, PBMC were stimulated with media alone, PPDb or rESAT-6:CFP10 for 16h. **(A)** Relative contribution of Tcm, Tem, and T effector cells to IFN-γ production in response to PPDb by long-term (i.e., 14-day) (left) and *ex vivo* (i.e., 16 h) (right) cultures did not differ between *M*. *bovis* 95–1315 (solid) or *M*. *bovis* 10–7428 (dashed) infection groups for any of the phenotypes (Two-way ANOVA, Šídák’s multiple comparison post-test). **(B)** Relative distribution of Tcm, Tem and T effector CD4^+^ cells in response to PPDb. (mean ± SEM, *P < 0.05, **P < 0.01; n = 8, Two-way ANOVA, Šídák’s multiple comparison post-test).
(TIF)

S5 Fig Representative cytometric plots of the long-term and short-term cultured proliferative responses to mycobacterial antigens by CD4, CD8 and γδ T cells. Long-term and short-term cultured PBMCs were analyzed ~ 7 weeks after aerosol challenge with virulent *M*. *bovis*. Long-term cells consist of PBMC from *M*. *bovis* infected cattle cultured in the presence of rAg85A, rTB10.4, rESAT-6:CFP10 and PPDb for 13 days and then CellTrace violet-stained and re-stimulated with either rESAT-6:CFP10, PPDb or medium in the presence of fresh autologous adherent cells for an additional six days. Short-term cells consist of CellTrace violet-stained PBMC from *M*. *bovis* infected cattle cultured for six days in the presence of rESAT-6:CFP10, PPDb or medium.
(TIF)
