# Supplementary figures and images for: Characterization of Effector and Memory T Cell Subsets in the Immune Response to Bovine Tuberculosis in Cattle
Source: PLoS One. 2015 Apr 16;10(4):e0122571. doi: 10.1371/journal.pone.0122571 (PMC4400046; doi:10.1371/journal.pone.0122571)

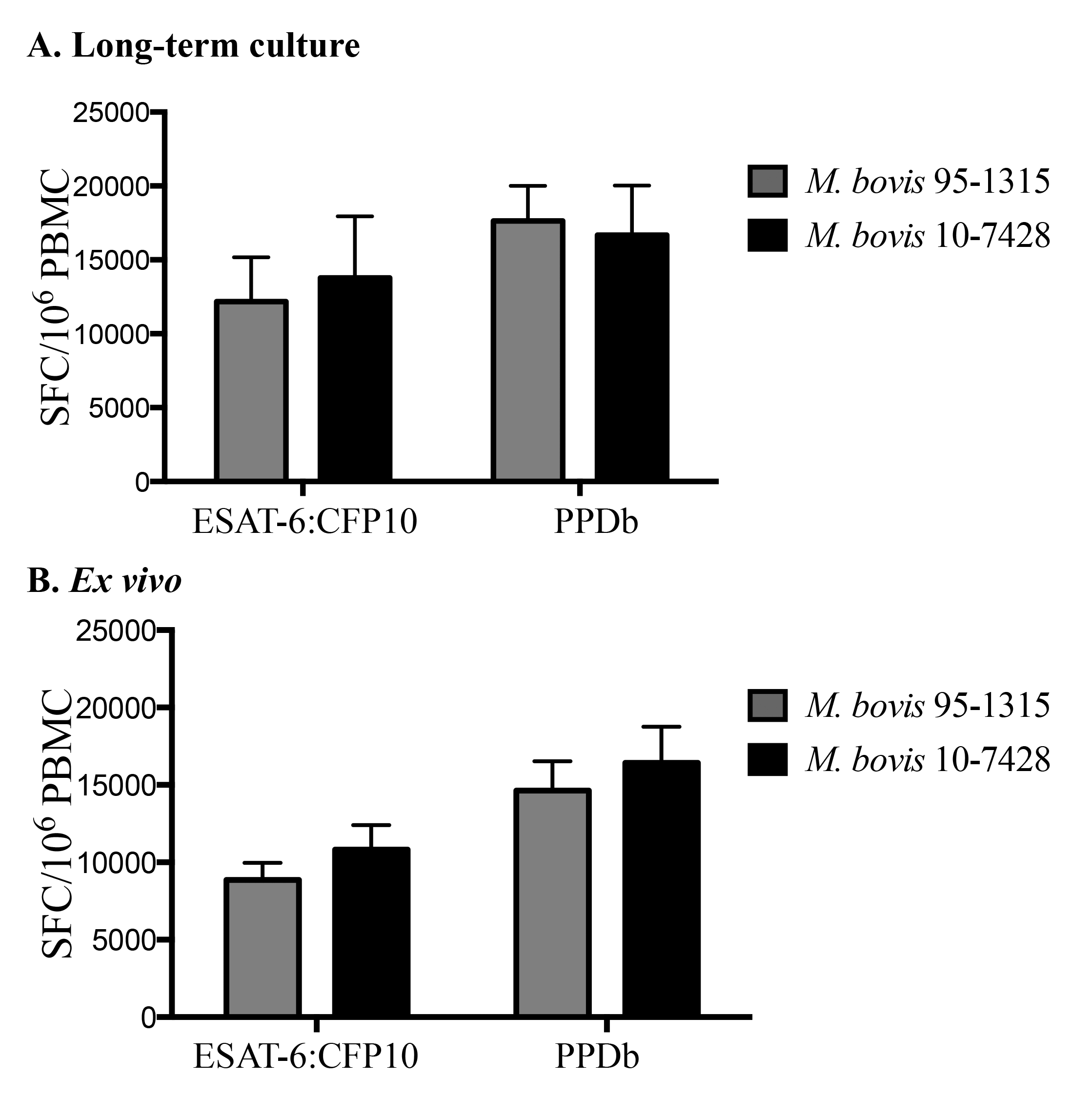

Supplement: S1 Fig — Cultured ELISPOT analysis was performed ~3 weeks after challenge with virulent M. bovis. Long-term cultured cells were generated by stimulating PBMC with a cocktail of rAg85A (1 μg/ml), rTB10.4 (1 μg/ml), and rESAT-6:CFP10 (1 μg/ml) antigens as well as PPDb (5 μg/ml) for 13 days followed by transfer to ELISPOT plates with APCs and addition of either rESAT-6:CFP10, PPDb or medium alone. For the ex vivo response freshly isolated PBMCs were stimulated with rESAT-6:CFP10, PPDb or medium alone for 16h. Medium control responses were subtracted from antigen-stimulated responses and results are presented as mean spot forming cells (SFC)/million cells (± SEM, n = 8) for (A) long-term culture or (B) ex vivo conditions. Responses did not differ between M. bovis 95–1315 and M. bovis 10–7428 infection groups (Two-way ANOVA, followed by Tukey’s multiple comparison). (TIF) [file pone.0122571.s001.tif]

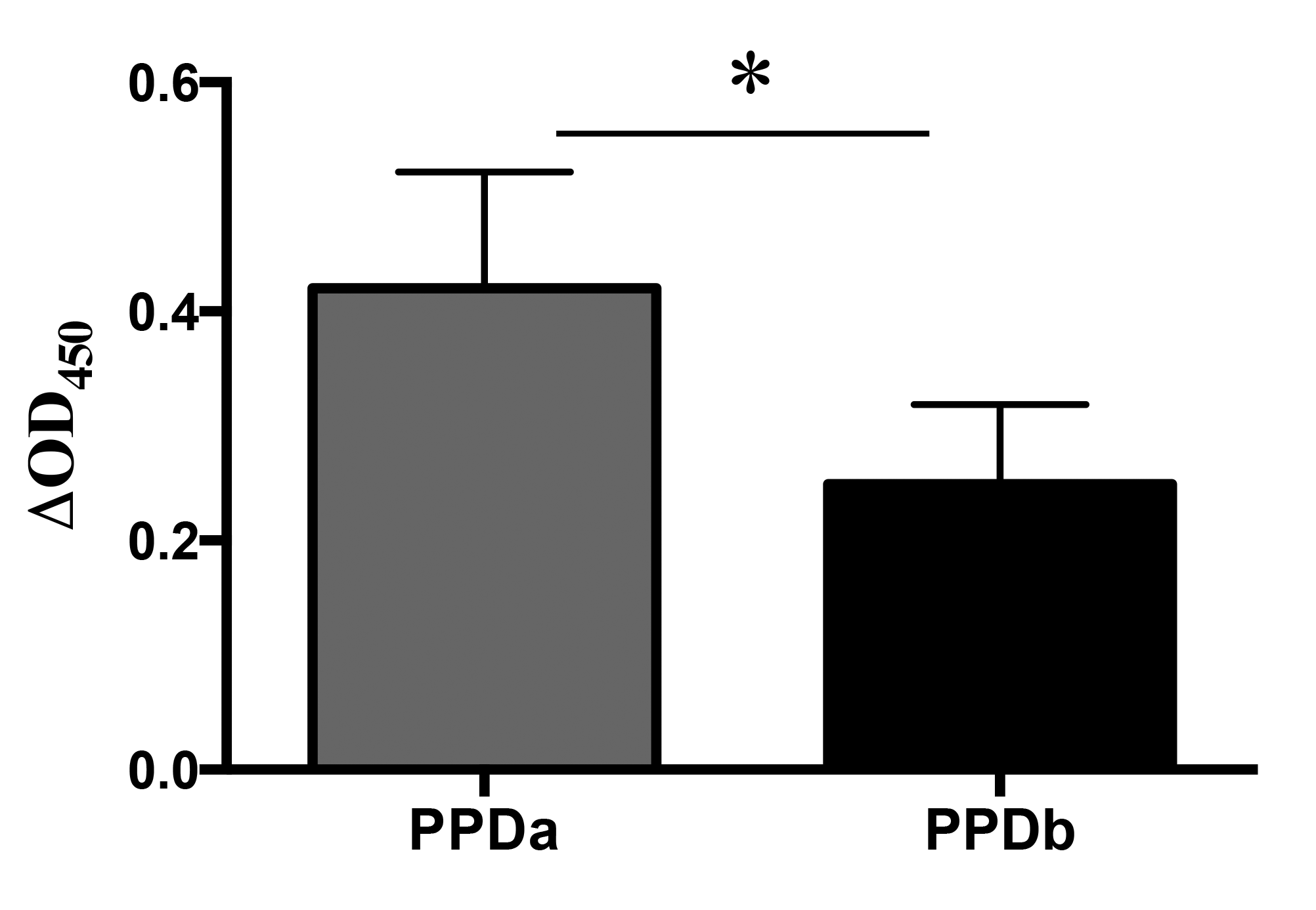

Supplement: S2 Fig — Responses to PPDa and PPDb prior to challenge were examined using a commercial IFN-γ release assay (i.e., Bovigam, Prionics Ag, Schlieren, Switzerland) according to manufacturer instructions. Briefly, duplicate 250l heparinised whole blood aliquots were distributed in 96-well plates with PPDb (10g/ml, Prionics Ag), PPDa (10g/ml, Prionics Ag), or no antigen and incubated at 39°C/5% CO2 for 20 hours. IFN-γ concentrations in stimulated plasma were determined using a commercial ELISA-based kit (Bovigam, Prionics Ag). Absorbencies of standards (recombinant bovine IFN-γ; Endogen, Rockford, IL) and test samples were read at 450 nm using an ELISA plate reader (Molecular Devices, Menlo Park, CA). Duplicate samples for individual treatments were analyzed and data presented as optical densities at 450 nm of the response to PPDb or PPDa minus the response to no-antigen (mean ± SEM). *Response to PPDa exceeded (P < 0.05, n = 24, paired Student's t-test) the response to PPDb. (TIF) [file pone.0122571.s002.tif]

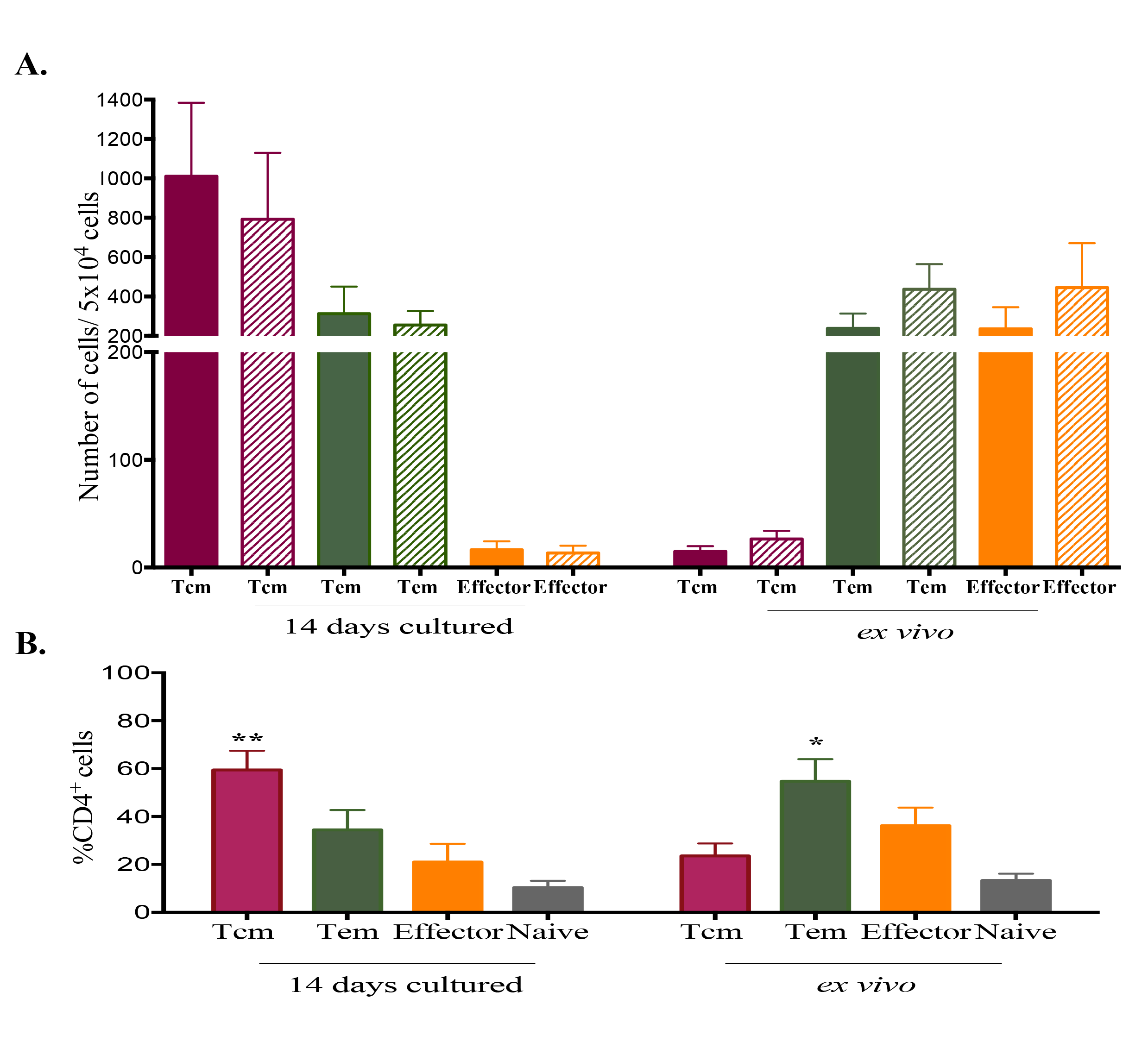

Supplement: S3 Fig — (A) Identification of memory subsets in the human peripheral blood based on the expression of CD45R0, CCR7, CD62L. (B) The differentiation of T cells occurs simultaneously with changes in cell functions. Adapted from Mahnkea et al. [46] (TIF) [file pone.0122571.s003.tif]

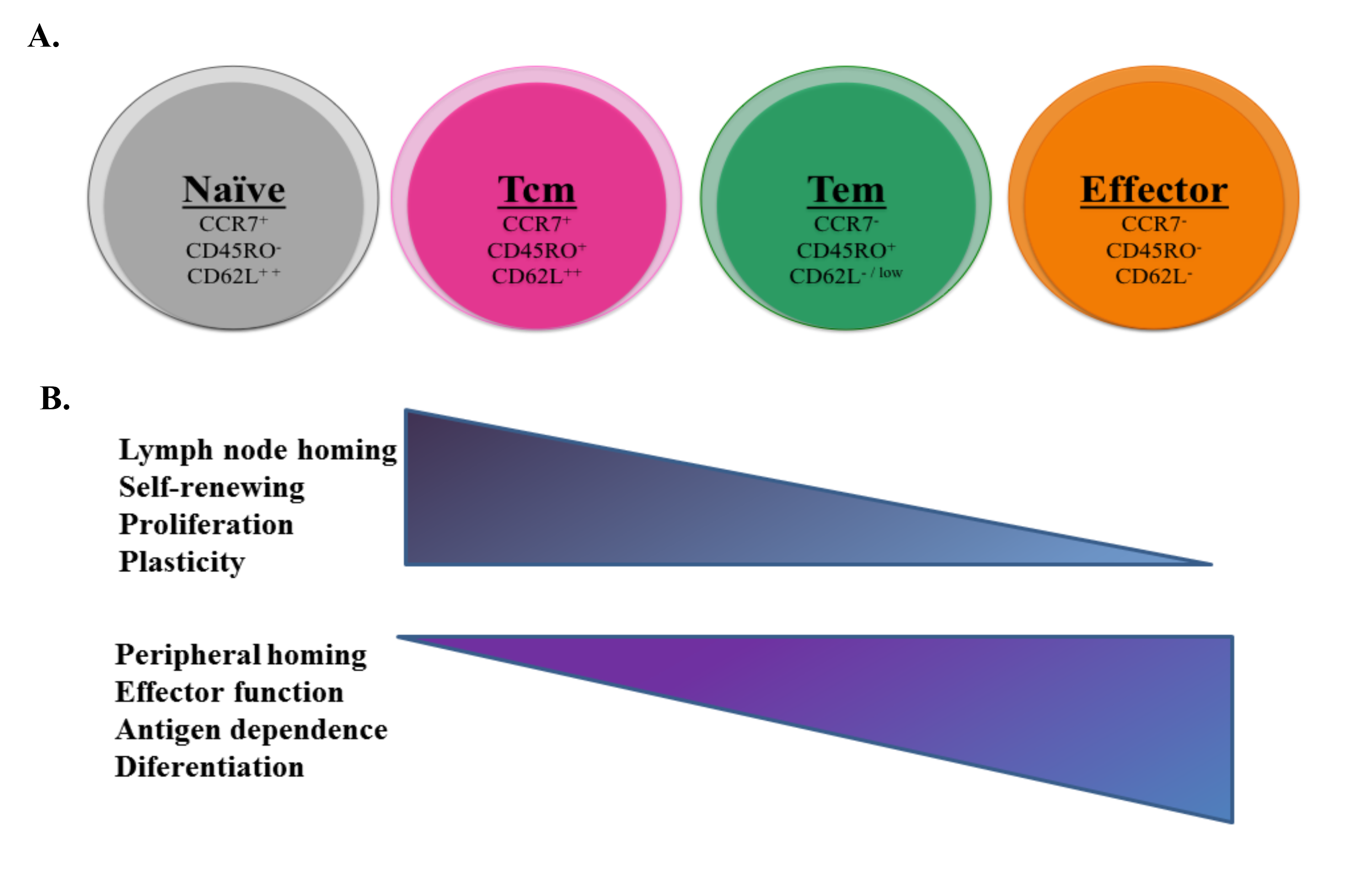

Supplement: S4 Fig — Peripheral blood mononuclear cells were isolated from calves ~ 8 weeks after challenge with virulent M. bovis (n = 8). Cells were stimulated with a cocktail of rAg85A (1 μg/ml), rTB10.4 (1 μg/ml), and rESAT-6:CFP10 (1 μg/ml) as well as PPDb (5 μg/ml) for 13 days followed by transfer to 96 well round bottom plates with APCs and addition of media alone, PPDb or rESAT-6:CFP10 for an additional 16h. For ex vivo culture, PBMC were stimulated with media alone, PPDb or rESAT-6:CFP10 for 16h. (A) Relative contribution of Tcm, Tem, and T effector cells to IFN-γ production in response to PPDb by long-term (i.e., 14-day) (left) and ex vivo (i.e., 16 h) (right) cultures did not differ between M. bovis 95–1315 (solid) or M. bovis 10–7428 (dashed) infection groups for any of the phenotypes (Two-way ANOVA, Šídák’s multiple comparison post-test). (B) Relative distribution of Tcm, Tem and T effector CD4+ cells in response to PPDb. (mean ± SEM, *P < 0.05, **P < 0.01; n = 8, Two-way ANOVA, Šídák’s multiple comparison post-test). (TIF) [file pone.0122571.s004.tif]

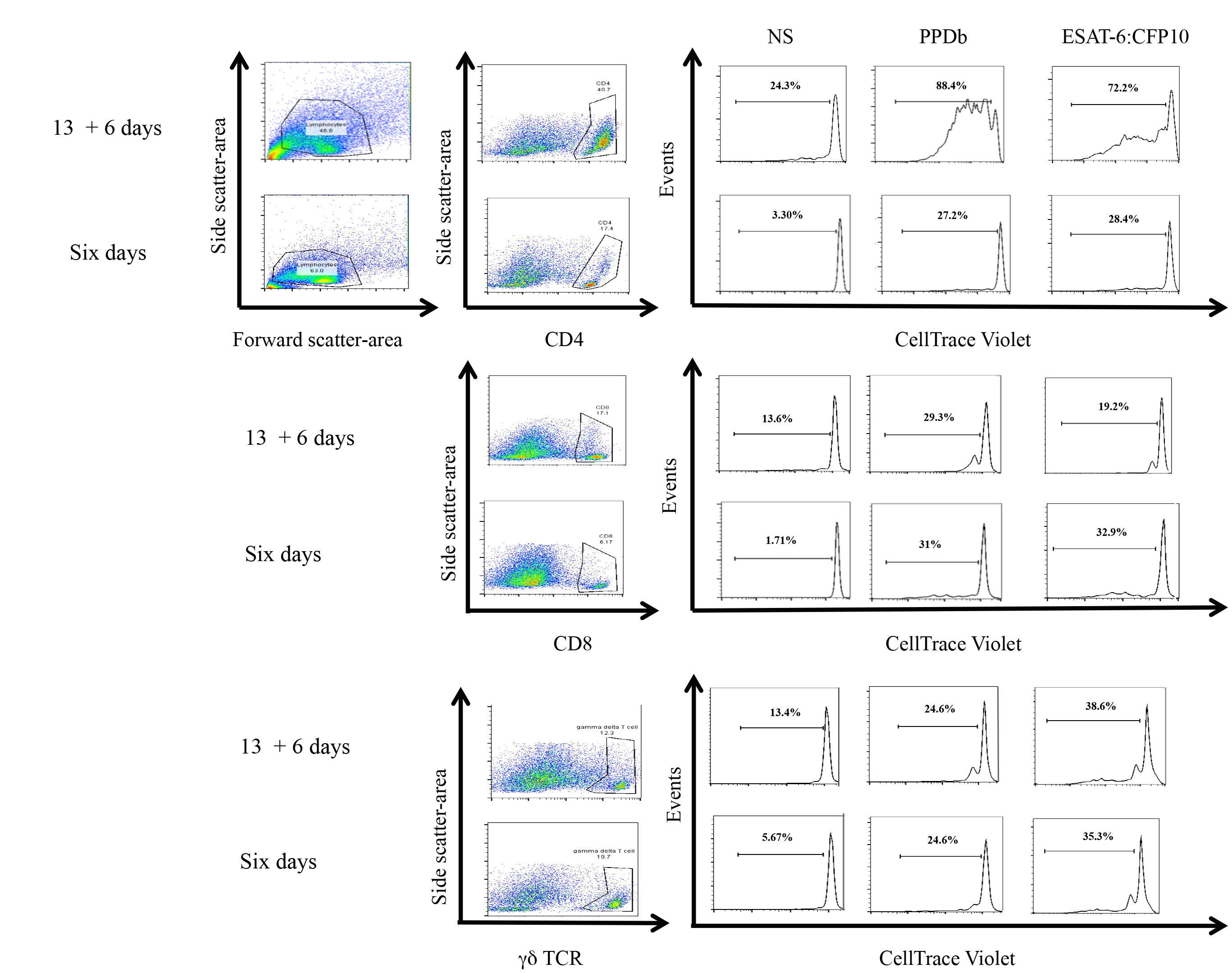

Supplement: S5 Fig — Long-term and short-term cultured PBMCs were analyzed ~ 7 weeks after aerosol challenge with virulent M. bovis. Long-term cells consist of PBMC from M. bovis infected cattle cultured in the presence of rAg85A, rTB10.4, rESAT-6:CFP10 and PPDb for 13 days and then CellTrace violet-stained and re-stimulated with either rESAT-6:CFP10, PPDb or medium in the presence of fresh autologous adherent cells for an additional six days. Short-term cells consist of CellTrace violet-stained PBMC from M. bovis infected cattle cultured for six days in the presence of rESAT-6:CFP10, PPDb or medium. (TIF) [file pone.0122571.s005.tif]
